# Tau: A Signaling Hub Protein

**DOI:** 10.3389/fnmol.2021.647054

**Published:** 2021-03-19

**Authors:** Rebecca L. Mueller, Benjamin Combs, Mohammed M. Alhadidy, Scott T. Brady, Gerardo A. Morfini, Nicholas M. Kanaan

**Affiliations:** ^1^Department of Translational Neuroscience, Michigan State University, Grand Rapids, MI, United States; ^2^Neuroscience Program, Michigan State University, East Lansing, MI, United States; ^3^Department of Anatomy and Cell Biology, The University of Illinois at Chicago, Chicago, IL, United States; ^4^Marine Biological Laboratory, Woods Hole, MA, United States; ^5^Hauenstein Neuroscience Center, Mercy Health Saint Mary’s, Grand Rapids, MI, United States

**Keywords:** tauopathy, kinase, phosphatase, scaffold protein, axon, synpase, nucleus, oligodendrocyte

## Abstract

Over four decades ago, *in vitro* experiments showed that tau protein interacts with and stabilizes microtubules in a phosphorylation-dependent manner. This observation fueled the widespread hypotheses that these properties extend to living neurons and that reduced stability of microtubules represents a major disease-driving event induced by pathological forms of tau in Alzheimer’s disease and other tauopathies. Accordingly, most research efforts to date have addressed this protein as a substrate, focusing on evaluating how specific mutations, phosphorylation, and other post-translational modifications impact its microtubule-binding and stabilizing properties. In contrast, fewer efforts were made to illuminate potential mechanisms linking physiological and disease-related forms of tau to the normal and pathological regulation of kinases and phosphatases. Here, we discuss published work indicating that, through interactions with various kinases and phosphatases, tau may normally act as a scaffolding protein to regulate phosphorylation-based signaling pathways. Expanding on this concept, we also review experimental evidence linking disease-related tau species to the misregulation of these pathways. Collectively, the available evidence supports the participation of tau in multiple cellular processes sustaining neuronal and glial function through various mechanisms involving the scaffolding and regulation of selected kinases and phosphatases at discrete subcellular compartments. The notion that the repertoire of tau functions includes a role as a signaling hub should widen our interpretation of experimental results and increase our understanding of tau biology in normal and disease conditions.

## Introduction

In 1975, the microtubule (MT)-associated protein tau was identified by the Kirschner laboratory as a protein that co-purified with tubulin and that significantly enhanced MT assembly *in vitro* (Weingarten et al., 1975; Witman et al., 1976; Cleveland et al., 1977a,b). These findings, together with the widespread expression of tau in the nervous system and its presence in axons, led to the presumption that tau was needed to stabilize microtubules in the axon, which has continued to be the most common description of tau function. Curiously, the generation of tau-null mice fell short of broadly changing this initial perception, despite their modest phenotype with advancing age and largely normal appearance of their nervous system (Harada et al., 1994; Dawson et al., 2001).

Several independent reports also showed that tau is the main constituent of paired helical filaments, a major histopathological hallmark of neurons affected in a group of neurodegenerative disorders termed tauopathies, which includes Alzheimer’s disease (AD; Grundke-Iqbal et al., 1986; Ihara, 1986; Wood et al., 1986; Kosik et al., 1988). In the mid-90s, strong genetic evidence linking mutations in the *MAPT* gene to inheritable forms of tauopathies indicated that altered tau function contributes to the pathogenesis of these diseases (Spillantini and Goedert, 1998). Also, amyloid-β neurotoxicity was reported to depend, at least in part, on the presence of tau protein (Rapoport et al., 2002). Thus, earlier work suggested a role for tau in stabilizing the MT cytoskeleton, further implicating this protein as an active contributor to neurodegenerative disease pathogenesis.

Concurrently, a significant body of experimental evidence gradually emerged suggesting that the repertoire of physiological tau functions might extend well beyond the regulation of MT stability. For example, early immunohistochemical studies documented tau localization at the leading growth cone of developing neurites, a compartment largely devoid of stable MTs (DiTella et al., 1994; Brandt et al., 1995; Black et al., 1996; Liu et al., 1999). Also, tau is localized within dendrites and somata of neuronal cells, and within compartments featuring scarce MTs, such as the nucleus and the subcortical cytoskeleton immediately adjacent to the inner plasma membrane (Papasozomenos and Binder, 1987; Loomis et al., 1990; Brandt et al., 1995). Although these observations strongly implied tau functions unrelated to MT stabilization, research efforts focused on unveiling such roles have remained relatively limited. Illuminating compartment-specific functions of tau would significantly advance our knowledge of its normal physiology and provide critical clues on the pathobiological roles played by this protein.

Scaffold proteins are important regulators of many intracellular signaling pathways. They often interact with various signaling proteins, coordinating their activities and localizing them to discrete cellular compartments or structures (Lester and Scott, 1997; Shaw and Filbert, 2009; Buday and Tompa, 2010). Interestingly, tau was reported to interact with many signaling proteins serving a wide variety of cellular functions (Morris et al., 2011; Sinsky et al., 2020). Notably, among tau interactors are a wide variety of kinases and phosphatases including protein phosphatase 1 (PP1), protein phosphatase 2A (PP2A), glycogen synthase kinase 3β (GSK3β), cyclin-dependent protein kinase 5 (CDK5), src-family kinases (cSrc, Fgr, Fyn, and Lck), as well as other proteins with established roles in signaling pathways including growth factor receptor-bound protein 2 (Grb2), p85α, and PLCγ (Liao et al., 1998; Sontag et al., 1999, 2012; Sun et al., 2002; Morris et al., 2011). Independently, other microtubule-associated proteins were found to interact with protein kinases. Among those, MAP2 interacts with protein kinase A (PKA) and acts as a PKA-anchoring protein (AKAP) that localizes this kinase in dendrites (Carnegie and Scott, 2003; Zhong et al., 2009).

Scaffold proteins commonly exhibit a remarkable degree of conformational flexibility, a feature that facilitates their dynamic interaction with multiple binding partners (Gunasekaran et al., 2003; Tompa and Fersht, 2009). Such flexibility represents another characteristic of tau documented by extensive structural and biophysical studies (Mandelkow and Mandelkow, 2012; Sabbagh and Dickey, 2016; Stern et al., 2017). Indeed, intrinsically disordered proteins, including tau, are known for their unique structural plasticity, conformational adaptability, and binding promiscuity enabling their involvement in diverse signaling roles (Uversky, 2015; Brandt et al., 2020). Interestingly, there is a clear evolutionary increase in disorder within the amino-terminal region of tau that likely enabled the development of novel protein-protein interactions (Trushina et al., 2019). It is noteworthy that, despite being categorized as an intrinsically disordered protein, tau is known to adopt specific folded conformations as a soluble protein (e.g., global hairpin folding; Jeganathan et al., 2006) and in the context of pathology (as identified by conformation-specific antibodies such as Alz50 or MC1; Wolozin et al., 1986; Carmel et al., 1996; Jicha et al., 1997, 1999). Some studies estimated the majority of tau is bound to MTs at any given moment, despite its dynamic interaction with MTs (~40 ms residence time *in vitro*). Such dynamics likely impact tau’s availability to interact with kinases and phosphatases on and off the MT surface (Weissmann et al., 2009; Janning et al., 2014). Based on its widespread subcellular distribution, diverse interactome and highly dynamic structural properties, tau could be considered a scaffold protein or “signaling hub” for the regulation of phosphorylation-based pathways in discrete subcellular compartments, as previously suggested (Lee et al., 1998; Morris et al., 2011; Kanaan et al., 2012; Götz et al., 2013; Sotiropoulos et al., 2017).

The goal of this review is to present an overview of published work supporting the role of tau in numerous cellular processes through interactions with and/or regulation of selected kinases and phosphatases. This overview includes the description of a PP1-GSK3β pathway by which tau regulates the transport of vesicles and other cellular organelles in axons. We also describe evidence supporting the role of tau on the regulation of Fyn kinase at post-synaptic compartments and oligodendrocyte processes, which impacts synaptic function and myelination, respectively. We discuss the potential roles of tau on the modulation of signaling pathways in nuclear and synaptic compartments. We succinctly review emerging evidence suggesting a role of tau on the modulation of phosphorylation-based pathways elicited by insulin and neurotrophic factors. Finally, based on strong genetic and experimental evidence linking pathological forms of tau to tauopathies, we examine evidence demonstrating deregulation of these pathways by pathogenic forms of tau.

## Tau Regulation of Signaling Pathways in Axons

The movement of membrane-bound organelles (MBOs) along axons, a cellular process referred to as fast axonal transport (FAT), is critical for appropriate maintenance of neuronal connectivity and survival (Black, 2016). The correct functionality of specialized axonal subcompartments, including nodes of Ranvier and pre-synaptic terminals, critically depends on the sustained supply of MBOs moving *via* anterograde FAT (aFAT; from the neuronal soma towards the cell periphery). Conversely, neuronal survival and homeostasis depend on retrograde FAT (rFAT) of specialized MBOs bearing neurotrophins and degraded materials from peripheral compartments towards the neuronal soma. In the mature brain, aFAT and rFAT are mainly driven by the multi-subunit motor proteins kinesin-1 (conventional kinesin) and cytoplasmic dynein, respectively. Both directions of FAT are regulated, in part, by signaling pathways controlling phosphorylation of these microtubule-based motors (Gibbs et al., 2015; Brady and Morfini, 2017). Highlighting the critical importance of FAT regulation to neuronal function, genetic and experimental evidence has linked deficits in FAT, synaptic dysfunction, and axonal pathology to numerous neurodegenerative diseases, including tauopathies (Morfini et al., 2009; Kanaan et al., 2013; Kneynsberg et al., 2017).

**Figure 1:**
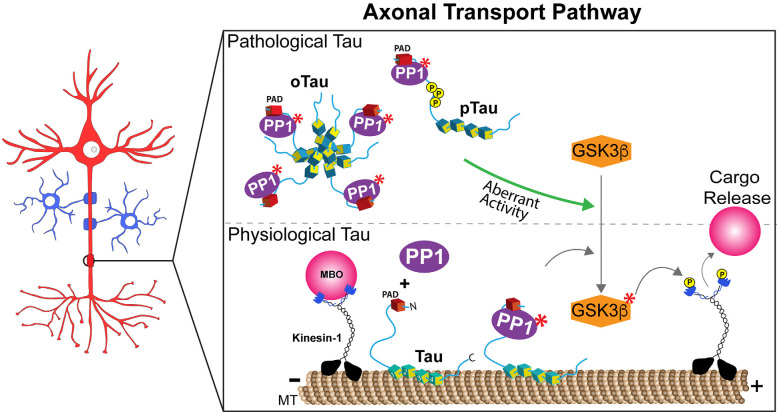
Tau regulates anterograde fast axonal transport through a PP1-GSK3β pathway. **Lower panel**: Exposure of a phosphatase activating domain (PAD, red domain) located at the N-terminus of tau causes activation of protein phosphatase 1 (PP1, in purple). Active PP1 (labeled by red asterisks) dephosphorylates and activates glycogen synthase kinase 3β (GSK3β). Active GSK3β (labeled by red asterisks) phosphorylates light chain subunits of kinesin-1, an event that promotes the release of membrane-bound organelle (MBO) cargoes transported by this major motor protein. Spatially discrete post-translational modifications in tau that either promote (i.e., phosphorylation events in the proline-rich region) or prevent (i.e., N-terminal phosphorylation) PAD exposure may thus allow the localized delivery of selected MBOs at functionally distinct axonal subdomains, such as *en passant* pre-synaptic terminals or nodes of Ranvier under normal physiological conditions. Although depicted bound to microtubules (MT), tau’s dynamic interaction with MTs in normal conditions likely provides opportunities for tau to interact with PP1 (and/or other enzymes) on or off the MT surface. **Upper panel**: By extension, aberrant PAD exposure associated pathological forms of tau such as aggregates/oligomers (oTau), mutant tau, and selected pathological post-translational modifications (e.g., phosphorylation, pTau), may disrupt homeostatic maintenance of this process.

A PP1-GSK3β pathway was previously defined that selectively regulates kinesin-1-based aFAT (Morfini et al., 2004). This pathway involved PP1 activation, resulting in dephosphorylation and activation of GSK3β, GSK3β-mediated phosphorylation of kinesin-1 light chain subunits, and detachment of this motor protein from its transported MBO cargoes (Ratner et al., 1998; Morfini et al., 2002, 2004; Figure 1, lower panel). Tau’s role in regulating this pathway was identified by experiments in isolated squid axoplasm, a plasma membrane-free *ex vivo* preparation uniquely suited for the study of FAT mechanisms (Gibbs et al., 2015; Song et al., 2016). Perfusion of squid axoplasm with physiological levels of recombinant human wild-type tau monomers did not affect aFAT or rFAT. In contrast, perfusion of axoplasm with disease-related forms of tau, including mutant and aggregated forms, selectively inhibited aFAT in a manner dependent on PP1 and GSK3β activities (LaPointe et al., 2009; Kanaan et al., 2011, 2012; Cox et al., 2016). Providing a mechanistic basis for these results, deletion experiments mapped a specific motif in the extreme N-terminus of tau, termed the Phosphatase Activating Domain (PAD), which was necessary and sufficient to activate the PP1-GSK3β pathway in axons (Kanaan et al., 2011, 2012). Specifically, aggregates composed of tau in which PAD was deleted did not inhibit aFAT, whereas perfusion with a synthetic PAD peptide (amino acids 2-18) sufficed to inhibit aFAT in the isolated axoplasm preparation (Kanaan et al., 2011). The observation that wild-type, soluble monomeric tau did not affect FAT despite featuring the PAD was consistent with studies showing that soluble tau monomers adopt a globally folded “paperclip” conformation, in which the C-terminus interacts with the MTBR, and the N-terminus interacts with the C-terminus (Jeganathan et al., 2006). Thus, folding of tau into the paperclip or paperclip-like conformations likely reduces exposure and accessibility of PAD in the soluble monomeric state. In support, monomeric tau lacking amino acids 144-273, a region that confers flexibility necessary for the N-terminus to fold into the paperclip conformation, inhibited aFAT when perfused in the isolated squid axoplasm preparation (Kanaan et al., 2011). Relevant to disease pathogenesis, truncation of amino acids 144-273 in tau is caused by a mutation in *MAPT* associated with an inherited tauopathy (Rovelet-Lecrux et al., 2009). Collectively, the findings above revealed a mechanism where various pathological modifications, including selected mutations, truncations, and aggregation, induced conformational changes in tau that promoted PAD exposure, activation of a PP1-GSK3β pathway, and inhibition of aFAT (Figure 1, upper panel).

In addition to mutations and aggregation, some phosphorylation events in tau were also found to promote PAD exposure, activation of the PP1-GSK3β pathway, and FAT inhibition. For example, the paperclip conformation is disrupted in tau monomers featuring pseudophosphorylation of three residues within the AT8 antibody epitope (i.e., S199, S202, and T205; pAT8-Tau), causing aberrant exposure of the N-terminus (Jeganathan et al., 2006; Kanaan et al., 2011). As expected, perfusion of squid axoplasm with pAT8-Tau monomers inhibited aFAT, an effect blocked by both PP1 and GSK3β inhibitors (Kanaan et al., 2011). In addition, aggregates composed of tau pseudophosphorylated at S422, a modification found in pre-tangle neurons affected early in AD, also inhibited both aFAT and rFAT when perfused in isolated axoplasm, but a potential involvement of PAD on these effects has not yet been evaluated (Tiernan et al., 2016). The highly dynamic conformational flexibility of tau, differentially impacted by phosphorylation of specific residues, might confer upon this protein its ability to work as a signaling hub and activate and/or modulate different signaling pathways (Smith et al., 2006; Kang et al., 2020). Collectively, these and other studies suggest a complex relationship between specific phosphorylation events in tau and their net impact on tau conformation, as well as the potential co-existence of additional biologically active domains in tau that, like PAD, may promote activation of other signaling pathways (Morris et al., 2020).

The ability of PAD to recruit and activate a PP1-GSK3β pathway close to microtubules bears important implications for our understanding of axon-specific functions of tau in normal and disease states (Figure 1). For example, spatially discrete phosphorylation events in tau that promote PAD exposure may facilitate the delivery of selected MBOs to their correct destinations (i.e., MBOs containing synaptic vesicle precursors to pre-synaptic terminals, or MBOs containing sodium channels at nodes of Ranvier; Morfini et al., 2001, 2016). Interestingly, phosphorylation of Y18 within PAD prevented the inhibitory effect of pathological tau species on aFAT (Kanaan et al., 2012), suggesting that phosphorylation of the Y18 residue by non-receptor tyrosine kinases might act as a negative feedback mechanism to prevent or downregulate PAD-dependent activation of the PP1-GSK3β pathway (Lee et al., 2004; Lebouvier et al., 2008; Lee and Leugers, 2012). Truncation of tau’s N-terminal region containing PAD by caspases or other proteases may represent an irreversible version of another negative feedback mechanism (Horowitz et al., 2004; Sengupta et al., 2006). Finally, the intriguing observation that tau phosphorylation at the Tau1 epitope (aa 192-204) is significantly lower in axons than in the soma and dendrites might relate to modulatory effects of this modification on global tau conformation, which in turn would promote or prevent activation of selected signaling pathways at discrete subcellular compartments (Papasozomenos and Binder, 1987; Mandell and Banker, 1995).

PAD’s contribution to neuronal pathology in the context of tauopathies is supported by immunochemical studies using the monoclonal antibodies tau N-terminal 1 and 2 (TNT1 and TNT2). These antibodies selectively recognize an epitope within PAD that is exposed in a conformation-dependent manner (Kanaan et al., 2011; Combs et al., 2016; Cox et al., 2016). Neurons featuring pre-tangle tau inclusions are immunoreactive to TNT antibodies across all Braak stages with relatively little reactivity in control cases and substantial reactivity in AD brains (Kanaan et al., 2011; Combs et al., 2016). TNT1/2 antibodies also robustly label tau inclusions in several non-AD tauopathies including progressive supranuclear palsy (PSP), corticobasal degeneration (CBD), Pick’s disease, and chronic traumatic encephalopathy (CTE; Kanaan et al., 2011, 2012; Combs et al., 2016; Cox et al., 2016; Tiernan et al., 2016; Combs and Kanaan, 2017). Moreover, pathological forms of tau known to inhibit FAT including oligomers, AT8 phospho-tau, and tau phosphorylated at Ser422 are all recognized by TNT1 and TNT2 antibodies in the context of human tauopathies (Kanaan et al., 2011, 2012, 2016; Patterson et al., 2011; Combs et al., 2016; Cox et al., 2016; Tiernan et al., 2016; Morris et al., 2020). Overall, this body of work strongly supports a mechanism linking several specific pathological tau species to aberrant PAD exposure, abnormal activation of a PP1-GSKβ pathway, and deficits in FAT. Potential pathological consequences of tau-induced FAT deficits may include impaired delivery of synaptic vesicles and/or mitochondria to pre-synaptic compartments, which would promote synaptic dysfunction and degeneration (Kneynsberg et al., 2017; Pérez et al., 2018).

## Tau Modulation of Signaling Pathways Controlling Synaptic Function

Long-term, activity-dependent strengthening or weakening of synapses are referred to as long-term potentiation (LTP) or long-term depression (LTD), respectively (Lüscher and Malenka, 2012). The regulation of both LTP and LTD involves the coordinated modulation of several neurotransmitter receptors and their downstream signaling pathways. N-methyl-D-aspartate receptors (NMDARs) are major mediators of LTP, although some forms of LTP are NMDAR-independent and can involve metabotropic receptors, kainate receptors, and α-amino-3-hydroxy-5-methyl-4-isoxazolepropionic receptors (AMPARs; Bliss et al., 2018). One mechanism that underlies the sustained enhanced synaptic response in LTP is the insertion of α-amino AMPARs into the synaptic membrane (Bliss et al., 2018). Conversely, AMPAR endocytosis is a mechanism contributing to LTD (Lüscher and Malenka, 2012). Accumulating evidence suggests that, by regulating the activity of specific signaling proteins involved in these processes, tau may impact synaptic function in health and disease states.

Under normal conditions, tau undergoes activity-dependent translocation to excitatory post-synapses (Frandemiche et al., 2014), a location where tau interacts with Fyn kinase and other post-synaptic proteins, including PSD-95 and NMDARs (Ittner et al., 2010). Analysis of tau knockout mouse models suggested the involvement of tau in the regulation of LTP, LTD, or potentially both (Ahmed et al., 2014; Kimura et al., 2014; Regan et al., 2015). Interestingly, Regan et al. (2015) showed that hippocampal LTD requires phosphorylation of tau at S396 (a residue within the PHF-1 epitope; Kimura et al., 2014). While these studies provide evidence that tau modulates synaptic function, the precise mechanisms and/or requirement of tau for LTP and LTD requires further investigation. In the context of disease, other studies documented the effects of pathological tau species on synaptic function (Tracy and Gan, 2018; Hanger et al., 2019; Hill et al., 2020). Indeed, oligomeric tau, mutant forms of tau associated with inherited tauopathies, abnormally phosphorylated tau species, acetylated tau, and caspase-cleaved tau were shown to impair LTP and/or LTD function, promoting abnormal AMPAR trafficking through various mechanisms (Yoshiyama et al., 2007; Hoover et al., 2010; Warmus et al., 2014; Min et al., 2015; Fá et al., 2016; Tracy et al., 2016; Zhao et al., 2016; Puzzo et al., 2017; Ondrejcak et al., 2018). Below, we present a succinct discussion of mechanisms linking tau to the regulation of signaling pathways sustaining the functionality of pre- and post-synaptic compartments.

Several studies indicated that neural network excitability is promoted by tau overexpression and counteracted by tau reduction (Roberson et al., 2007; Miyamoto et al., 2017). These effects were linked to alterations in the subcellular localization and activity of Fyn, a member of the Src family of non-receptor tyrosine kinases highly enriched in brain tissue. Mice overexpressing Fyn showed NMDAR overactivation and excitotoxicity, highlighting a role for this kinase on synaptic function (Ittner et al., 2010; Xia et al., 2015). The projection domain of tau contains seven PXXP motifs, two of which can directly interact with the SH3 domain of Fyn (Umemori et al., 1992; Lee et al., 1998). Specifically, tau-Fyn interaction depends on prolines 213, 216, and 219 within the fifth and sixth PXXP motifs (Lau et al., 2016), although isoform-specific differences may exist (Bhaskar et al., 2005). Known tau modifications, such as phosphorylation in the N-terminal half of the protein (Bhaskar et al., 2005; Reynolds et al., 2008) and pathological aggregation (Fitzpatrick et al., 2017; Goedert and Spillantini, 2019), may alter tau conformation in the proline-rich region and block Fyn binding domains. Tau is not only a substrate of Fyn (Lee et al., 2004; Bhaskar et al., 2005), but also increases auto-phosphorylation and activation of this kinase (Sharma et al., 2007).

**Figure 2:**
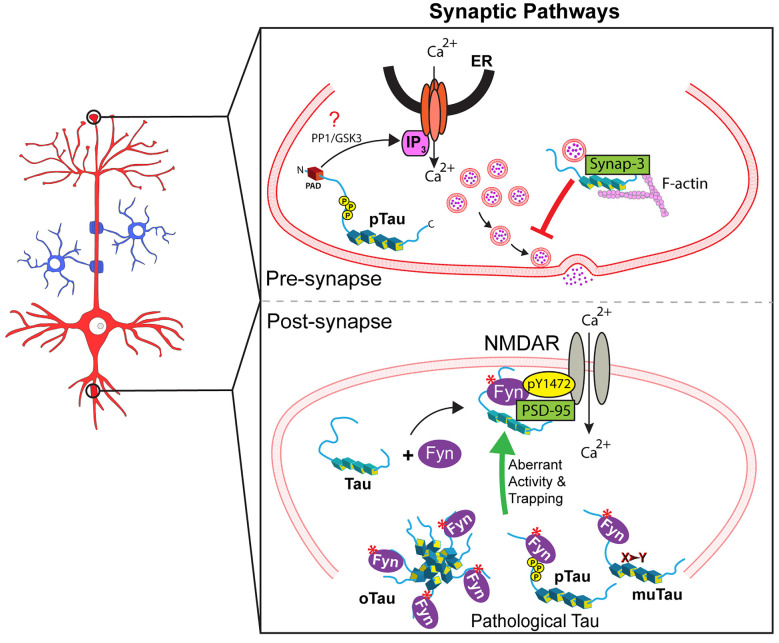
Tau regulates several signaling synaptic pathways. **Upper panel**: At the pre-synaptic compartment, tau undergoes phosphorylation at the AT8 epitope (pTau). This event promotes PAD exposure, which in turn increases Ca^2+^ release from intracellular stores and reduces neurotransmitter release in an IP_3_-dependent manner, which might involve PP1 and/or GSK3 (indicated by a red question mark). Also, pathological forms of tau reportedly restrict vesicle mobility through scaffold interactions with the vesicular protein synaptogyrin-3 and F-actin. **Lower panel**: Tau localizes Fyn to the post-synapse allowing Fyn to phosphorylate NMDAR2b at Y1472, which leads to NMDAR stabilization *via* interaction with PSD-95. Pathological forms of tau including aggregated/oligomeric tau (oTau), specific phosphorylated forms of tau (pTau), or mutant tau (muTau) can affect tau-Fyn binding, ultimately leading to aberrant synaptic activity by promoting increased trapping of Fyn in dendritic spines.

Several lines of evidence support the tau-based localization of Fyn to the postsynaptic compartment. For example, reduced levels of Fyn in post-synapses were observed in tau knockout mice (Ittner et al., 2010). Also, a recent study used single-molecule tracking to show that tau knockdown increased the mobility of Fyn in dendritic shafts (as opposed to spines), an effect rescued by tau expression (Padmanabhan et al., 2019). Tau-mediated localization of Fyn to post-synapses facilitated Fyn-mediated phosphorylation of the NMDAR subunit 2b at tyrosine 1472 (Nakazawa et al., 2001; Ittner et al., 2010). This phosphorylation event promotes stabilization of NMDARs by facilitating their interaction with the scaffolding protein PSD-95 (Grant et al., 1992; Suzuki and Okumura-Noji, 1995; Nakazawa et al., 2001; Roche et al., 2001; Rong et al., 2001). Conversely, reduced localization of Fyn to the post-synapse negatively impacted the stability of the NMDAR-PSD-95 complexes (Ittner et al., 2010). Collectively, these studies demonstrate that tau acts as a scaffold protein for Fyn in the post-synaptic compartment where it participates in NMDAR-mediated synaptic functions by modulating Fyn localization and/or activity (Figure 2).

Pathological forms of tau promote abnormalities in the Fyn-dependent control of synaptic function through a variety of mechanisms. Some tau mutants associated with inherited tauopathies displayed increased interaction with Fyn (Bhaskar et al., 2005), and other reports documented abnormalities in Fyn localization in mouse models of inheritable tauopathies. For example, recent characterization of Fyn nanoclustering in mouse hippocampal dendritic spines showed that P301L mutant tau trapped Fyn in the spines (Padmanabhan et al., 2019), a finding consistent with prior reports (Hoover et al., 2010; Ittner et al., 2010; Xia et al., 2015), and a role of tau as a scaffolding protein. Also, Hoover and colleagues documented increased localization of P301L tau in dendritic spines of rat cultured neurons compared to wild-type tau, and this finding was confirmed in living neurons of transgenic mice expressing mutant P301L tau (Tg4510 line; Hoover et al., 2010). Using overexpression experiments in cultured mouse hippocampal neurons, Xia et al. (2015) reported 5-fold higher levels of spine localization for P301L tau, compared to wild-type tau. Both groups also showed that phosphorylation of tau at specific sites influenced its targeting to dendritic spines (Hoover et al., 2010; Xia et al., 2015). Together, these studies suggest that mutations and specific phosphorylation events may affect tau’s ability to localize to post-synapses and/or bind and activate Fyn, an event that might contribute to the synaptic dysfunction phenotype observed in tauopathies.

In recent years, additional experimental evidence indicated a potential role for tau in pre-synaptic function through PP1 regulation. For example, injection of wild-type tau in pre-synaptic terminals of the squid giant synapse increased Ca^2+^ release and reduced neurotransmitter release. This effect was associated with rapid phosphorylation of tau at the AT8 epitope (Moreno et al., 2016), an event that promotes increased exposure of PAD (Kanaan et al., 2011). Accordingly, co-injection of wild-type tau with TNT1 antibody prevented its toxic effect on synaptic transmission (Moreno et al., 2016). Independently, other studies revealed increased levels of phosphorylated tau associated with synaptic vesicles in AD tissue, and mutant forms of tau (e.g., P301L, P301S, R406W, or V337M tau) interfered with pre-synaptic function in multiple models of tauopathy (Zhou et al., 2017; McInnes et al., 2018). Mechanistically, these effects were linked to abnormal scaffolding behavior of tau, which restricted vesicle mobility through interactions with synaptogyrin-3 and actin (Zhou et al., 2017; McInnes et al., 2018). Collectively, these findings suggest that tau regulates the functionality of pre- and post-synaptic compartments through the modulation and/or localization of kinases and phosphatases and that pathological forms of tau may alter such regulation through a variety of mechanisms (Figure 2).

## Tau Regulation of Signaling Pathways in The Nucleus

**Figure 3:**
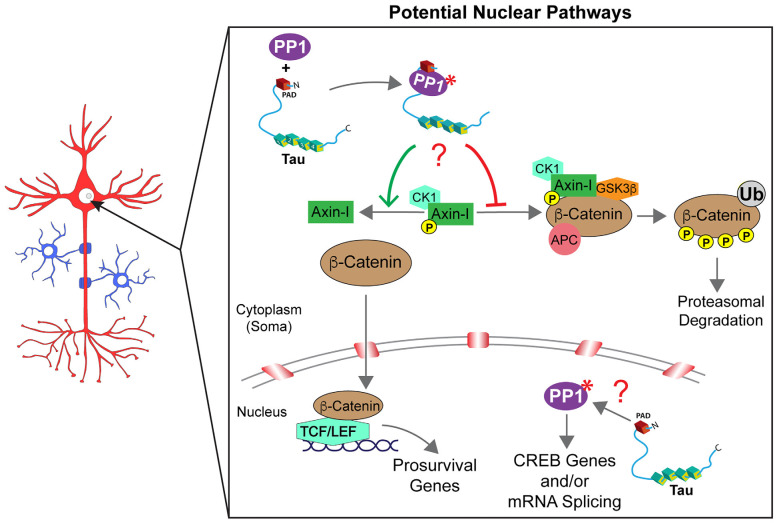
Tau may control multiple signaling pathways in the nucleus. β-catenin degradation/signaling is regulated by the assembly or disassembly of a cytoplasmic destruction complex [composed of glycogen synthase kinase-3β (GSK3β), casein kinase 1 (CK1), adenomatous polyposis coli (APC), and axin-I]. Whether β-catenin is free to translocate into the nucleus (anti-apoptotic conditions) or degraded in the cytoplasm through ubiquitination (Ub; in pro-apoptotic conditions) is driven, at least in part, by the phosphorylation status of axin-I. PP1 directly regulates axin-I phosphorylation with PP1-dependent dephosphorylation of axin-I preventing the formation of the destruction complex and favoring anti-apoptotic β-catenin signaling in the nucleus. Through interactions *via* its PAD domain, cytoplasmic tau may activate PP1 (marked by a red asterisk) and promote axin-I dephosphorylation (indicated by a red question mark), but this potential mechanism awaits further investigation. Similarly, the co-location of tau and PP1 within the nucleus suggests a potential role may exist for tau to modulate intra-nuclear PP1-dependent pathways such as cAMP response element-binding protein phosphorylation and signaling and/or mRNA splicing. However, future studies are needed to fully elucidate potential intra-nuclear tau signaling mechanisms (indicated by a red question mark).

By regulating the activity of PP1 and associated downstream signaling cascades, tau might regulate cellular processes in compartments beyond axons and synapses. In support, both tau and PP1 localize to the nucleus and soma (Rebelo et al., 2015; Maina et al., 2016). PP1 is known to modulate multiple signaling pathways (Rebelo et al., 2015), including a Wnt/β-catenin pathway that promotes cell survival (Chen et al., 2001; Voskas et al., 2010). This pathway involves phosphorylation-dependent β-catenin degradation in the cytoplasm by a β-catenin destruction complex composed of the proteins [GSK3β, casein kinase-I (CK-I), and adenomatous polyposis coli (APC)] assembled by the scaffolding protein axin-I. Disruption of this protein complex promotes the accumulation of dephosphorylated β-catenin in the cytoplasm and its subsequent translocation into the nucleus. Within the nucleus, β-catenin associates with T-cell factor/lymphoid enhancer-binding factor (TCF/LEF) DNA-binding proteins to stimulate the transcription of prosurvival genes (Chen et al., 2001; Voskas et al., 2010). Outside the nucleus, PP1 enhances β-catenin nuclear transcription signaling by dephosphorylating axin-I. This event prevents the formation of the cytoplasmic β-catenin destruction complex, leading to accumulation and nuclear translocation of β-catenin (Luo et al., 2007). Interestingly, expression of a specific tau isoform (2N4R) in HEK293 and N2a cells attenuated apoptotic responses elicited by GSK3β overexpression or by treatment with the kinase inhibitor staurosporine. This protective, prosurvival effect of 2N4R tau was associated with reduced phosphorylation and enhanced nuclear translocation of β-catenin in cells (Li et al., 2007). The physiological relevance of these data remains to be established, but it appears consistent with a potential mechanism where tau-mediated activation of PP1 in the cytoplasm would impact nuclear β-catenin signaling (Figure 3).

Early studies documenting localization of tau in the nucleus implied a potential role of this protein in the control of nuclear activities, but such roles remain largely elusive (Loomis et al., 1990; Brady et al., 1995; Thurston et al., 1996). More recently, various studies documented interactions between tau and nuclear resident proteins such as TIP5, and roles for tau in heterochromatin stability, repression of ribosomal DNA transcription, and protection of DNA from stress-induced damage were proposed (Li et al., 2007; Mansuroglu et al., 2016). Inside the nucleus, PP1 regulates signaling pathways involved in the control of gene transcription, splicing, and cell-cycle progression (Rebelo et al., 2015). For instance, PP1 decreased cAMP-dependent gene expression by dephosphorylating a specific residue (Ser133) in cAMP-responsive element-binding protein (CREB; Hagiwara et al., 1992). PP1 facilitates splicing of pre-mRNAs by dephosphorylating spliceosome-associated protein 155 (SAP155; Rebelo et al., 2015) *via* an interaction facilitated by a nuclear inhibitor of protein phosphatase 1 (NIPP1). Furthermore, PP1 activity is required for the successful execution of pre-mRNA splicing (Mermoud et al., 1992). Considering the nuclear localization of tau and the functional interaction between tau and PP1 elsewhere in neurons, it is reasonable to speculate that tau-mediated PP1 activation could regulate signaling pathways in this compartment. Specific mechanisms linking tau to nuclear processes and potential routes through which pathogenic tau species might impair such processes warrant further investigation.

## Tau Modulation of Signaling Pathways in Oligodendrocytes

**Figure 4:**
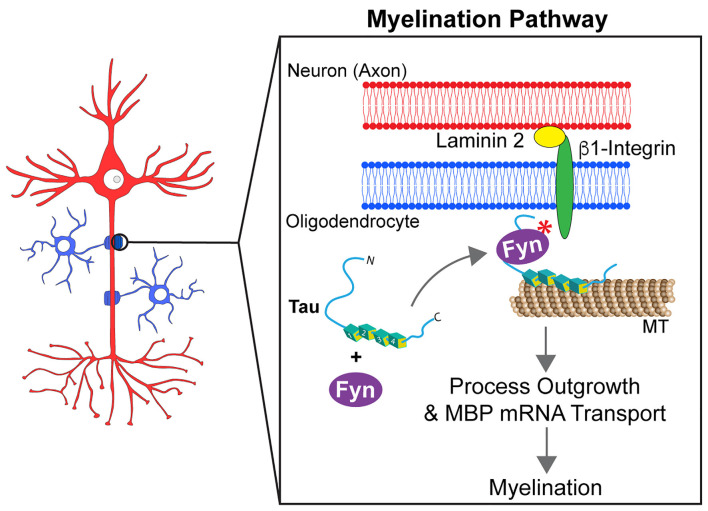
Tau modulates Fyn kinase pathways in myelinating oligodendrocytes. Myelination requires the outgrowth of oligodendrocyte processes and the formation of contact points between oligodendrocytes and axons. For this to occur, the mRNA of the myelin basic protein (MBP) needs to be targeted to those contact points. Tau acts as a scaffold protein linking Fyn kinase to the microtubule (MT) cytoskeleton of oligodendrocyte processes. Upon establishment of contact points at the neuron-glia interface, a signaling cascade involving tau and Fyn kinase is activated. That signaling pathway culminates into the rearrangement of the cell cytoskeleton that leads MBP mRNA to myelination sites and promotes the growth of oligodendrocyte processes.

Myelination of axons requires strict spatiotemporal regulation of myelin basic protein (MBP) mRNA translation at the axon-glia interface (White et al., 2008). In oligodendrocytes, MBP mRNA binds to heterogeneous nuclear ribonucleoprotein A2 (hnRNP A2) in mRNA transport granules, undergoing anterograde transport from the nucleus to oligodendrocyte processes in a translationally silent state (White et al., 2008; Müller et al., 2013). After initial contact points between oligodendrocytes and axons are established, Fyn kinase is activated by a β1-integrin-dependent signal transduction cascade. Consequently, Fyn phosphorylates hnRNP A2, reducing binding to MBP mRNA and dismantling RNA transport granules, which in turn allows local translation of MBP protein (White et al., 2008; Müller et al., 2013). The role of Fyn kinase in myelination and interactions between tau and Fyn suggest tau may impact this process in oligodendrocytes (Figure 4).

Tau is expressed at significant levels in oligodendrocytes (LoPresti, 2002, 2018) where it can form a complex with Fyn and α-tubulin and/or potentially actin (Lee et al., 1998; Klein et al., 2002). Seiberlich and colleagues reported that tau silencing prevented MBP mRNA transport in oligodendrocyte processes. Additionally, the absence of tau significantly reduced interaction between α-tubulin and Fyn (Seiberlich et al., 2015), suggesting that tau may act as a scaffold by linking Fyn to MTs in oligodendrocytes. Similar observations were made using a truncated form of wild-type tau (Δtau, amino acids 1-228), which maintains the interaction with Fyn but lacks the microtubule-binding repeat domain (Belkadi and LoPresti, 2008). CG-4 cells overexpressing Δtau displayed a shortening of oligodendrocyte processes along with mislocalization of Fyn to the cell body instead of processes. Furthermore, transplanting CG-4 cells that stably express Δtau into the spinal cord of a myelin-deficient rat model demonstrated a failure in myelination capacity (Belkadi and LoPresti, 2008). In transgenic mice expressing Δtau, oligodendrocytes displayed reduced myelination and the animals developed motor deficits (LoPresti, 2015). Together, these findings suggest that tau acts as a scaffold protein that targets Fyn kinase to MTs in oligodendrocyte processes, and that disruption of this function may impair mechanisms contributing to myelination (LoPresti, 2018).

## Tau Modulation of Signaling Pathways Activated by Insulin and Neurotrophin Receptors

Early studies involving immunostaining of cultured neurons documented a fraction of tau close to the plasma membrane of developing neurites, but functional roles at this subcellular location, largely devoid of MTs, remained elusive (Papasozomenos and Binder, 1987; Loomis et al., 1990; Brandt et al., 1995). Emerging evidence, succinctly discussed below, revealed modulatory effects of tau on signaling pathways triggered by insulin and neurotrophin receptors at this location, potentially shedding some light on this issue.

Signaling pathways activated by insulin play an important role in the maintenance of neuronal function and peripheral metabolism (Lee et al., 2016). Although typically associated with diabetes mellitus, alterations in insulin pathway signaling are also observed in AD, and a high rate of comorbidity is observed between AD and diabetes (Ott et al., 1999; Talbot et al., 2012). Insulin-related signaling is initiated upon insulin hormone binding to insulin receptors (IR). IRs are transmembrane tyrosine kinase receptors that phosphorylate themselves and other downstream substrates, including insulin receptor substrates (IRS1/2). IRS1/2 activate phosphatidylinositol 3-kinase (PI3K) which in turn phosphorylates phosphatidylinositol (3,4)-biphosphate (PIP_2_) to convert it into phosphatidylinositol (3,4,5)-triphosphate (PIP_3_). PIP_3_ recruits AKT to the cell membrane where it is activated and phosphorylates numerous kinase substrates (i.e., GSK3β, mTORC1, et cetera) that in turn regulate a multitude of cellular processes. Phosphatase and tensin homolog (PTEN) dephosphorylates PIP_3_, providing a mechanism to terminate insulin-dependent signaling.

**Figure 5:**
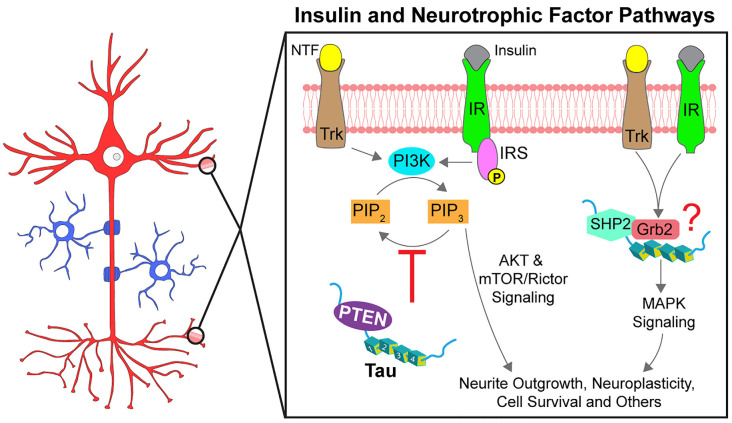
Tau promotes signal transduction of insulin and neurotrophic factor pathways. Neurotrophic factors (NTF) and insulin activate specific receptor tyrosine kinases (Trks and IRs, respectively). Following autophosphorylation, the receptors initiate signaling through multiple downstream pathways, two of which are partially modulated by tau. Activation of AKT signaling by PI3K is regulated by PIP_3_ levels, increased through phosphorylation by PI3K and decreased through dephosphorylation by PTEN. Tau reportedly binds to PTEN directly to inhibit its phosphatase activity (red inhibitory arrow), thereby promoting AKT activation. Tau can also promote MAPK signaling through an interaction with SHP2, a tyrosine phosphatase downstream of the receptor tyrosine kinases. Tau also interacts with the SH3 domain of Grb2, an activator of SHP2 but whether tau, SHP2, and Grb2 form a complex and the downstream effects are not known (indicated by a red question mark). Phosphorylation at specific residues may impact the modulatory role of tau on these pathways.

Several studies have linked insulin signaling pathway dysfunction to abnormal phosphorylation of tau (El Khoury et al., 2014). However, recent evidence also suggests that tau might modulate this pathway, a notion with important implications for normal and pathological tau functions. Genetic deletion of *MAPT* impaired insulin signaling by disrupting IRS-1 function and downstream PI3K-AKT-mTOR signaling (Marciniak et al., 2017). While these changes occurred in the brain, tau knockout mice also displayed peripheral hyperinsulinemia, glucose intolerance, and an attenuated food intake response to insulin treatment (Marciniak et al., 2017). Providing a potential molecular mechanism for this phenotype, tau interacted with PTEN *via* its proline-rich domain and reduced its lipid phosphatase activity (Marciniak et al., 2017; Tai et al., 2020). Accordingly, PTEN knockdown restored normal insulin signaling response in Tau KO mouse neurons suggesting that tau inhibits PTEN activity in physiological conditions. Extending these findings to tauopathy models, the accumulation of mutant P301L tau was associated with increased PTEN activation in the rTg4510 tau transgenic mouse model, which in turn contributed to synaptic degeneration (Benetatos et al., 2020). Together, these studies provide evidence that in normal conditions tau promotes PI3K-AKT signaling by inhibiting PTEN directly or indirectly, further suggesting that pathological modifications in tau that affect this interaction may promote synaptic dysfunction (Figure 5).

A wide range of extracellular and intracellular stimuli, including growth factors, hormones, and cytokines, trigger activation of mitogen-activated protein kinases (MAPKs) to regulate cell differentiation, survival, and death (Keshet and Seger, 2010). In neurons, activation of MAPKs by insulin and neurotrophic factor receptors (Trks) is linked to synaptic plasticity and survival, among many other cellular processes (Sun and Nan, 2017). Like IRs, Trks undergo tyrosine autophosphorylation upon binding to their neurotrophic ligand, an event that triggers interactions among Trks and various SH2 domain-containing effector proteins. Among these is Src homology phosphatase 2 (SHP2), a protein tyrosine phosphatase also known as PTPN11. SHP2 promotes activation of extracellular receptor kinases (ERKs), a specific subset of MAPKs critically involved in neurotrophin-based survival (Easton et al., 2006).

Various reports documented modulatory effects of tau on neurotrophin-based activation of MAPK signaling (Figure 5). For example, deletion of tau in PC12 cells impaired ERK activation and neurite outgrowth induced by nerve growth factor (NGF) stimuli (Leugers and Lee, 2010). Interestingly, the phosphorylation status of tau impacted the response. Tau phosphorylation at T231 potentiated NGF-induced activation of ERK1/2 kinases, whereas phosphorylation at S214 or the PHF1 site (S396/S404) inhibited this signaling (Leugers and Lee, 2010; Leugers et al., 2013). Additional work suggested that these effects might involve a direct, activating protein-protein interaction between tau and SHP2 (Kim et al., 2019). Tau and SHP2 interacted in pulldown assays and both proteins were identified as components of a larger protein complex using proximity ligation assays (Kim et al., 2019). Pseudophosphorylation at T231 in tau enhanced the interaction with SHP2 in cells compared to T231A tau (Kim et al., 2019). Evidence from human tissue studies suggests tau-SHP2 interactions are increased in mild cognitive impairment and in AD brains when compared to non-demented control brains (Kim et al., 2019). Additionally, tau interacts with the SH3 domain of an SHP2-activator protein, Grb2, but whether SHP2 and Grb2 interact simultaneously with tau and the functional implications of these interactions are not yet well-defined (Reynolds et al., 2008). Considered together, these data suggest that tau may modulate neurotrophin-induced activation of the MAPK signaling pathway, but whether such effects involve direct activation of SHP2 by tau directly or targeted localization to lipid rafts awaits further investigation.

## Conclusions

The long-held hypothesis that stabilization of MTs represents tau’s primary biological function is being increasingly challenged by a growing body of independent experimental evidence. Continuing to challenge this dogma will help expand our interpretation of such evidence and advance our understanding of tau biology in health and disease conditions. For example, the ability of tau to interact with MTs may reflect just one of several features enabling a much more diverse set of functions by this protein, such as the regulation of phosphorylation-based signaling pathways in axons and other MT-rich subcellular compartments. On the other hand, MT-independent interactions with additional proteins may facilitate similar functions in modulating pathways in other compartments featuring few or no MTs. This notion appears consistent with tau’s widespread subcellular distribution, its highly dynamic conformational flexibility, and the numerous kinase and phosphatase binding partners identified thus far. Based on these characteristics, we posit that tau functions as a signaling hub, regulating a wide set of cellular processes through the modulation of selected kinases and phosphatases. This function in turn appears to be impacted by specific modification events in tau, including mutations, phosphorylation, truncation, and aggregation, which could have important implications for physiological and disease states. A more dynamic perspective of tau as a multifaceted protein involved in the control of intracellular signaling may also provide a novel framework for the development of therapeutic approaches to treat tauopathies.

## Author Contributions

RM, BC, MA, SB, GM, and NK each contributed to writing and editing this review. All authors contributed to the article and approved the submitted version.

## Conflict of Interest

The authors declare that the research was conducted in the absence of any commercial or financial relationships that could be construed as a potential conflict of interest.

## References

[B1] Ahmed T. Van der Jeugd A. Blum D. Galas M. C. D’Hooge R. Buee L.. (2014). Cognition and hippocampal synaptic plasticity in mice with a homozygous tau deletion. Neurobiol. Aging 35, 2474–2478. 10.1016/j.neurobiolaging.2014.05.005 24913895

[B2] Belkadi A. LoPresti P. (2008). Truncated Tau with the Fyn-binding domain and without the microtubule-binding domain hinders the myelinating capacity of an oligodendrocyte cell line. J. Neurochem. 107, 351–360. 10.1111/j.1471-4159.2008.05600.x 18680553

[B3] Benetatos J. Bennett R. E. Evans H. T. Ellis S. A. Hyman B. T. Bodea L. G.. (2020). PTEN activation contributes to neuronal and synaptic engulfment by microglia in tauopathy. Acta Neuropathol. 140, 7–24. 10.1007/s00401-020-02151-9 32236736 PMC7300099

[B4] Bhaskar K. Yen S.-H. Lee G. (2005). Disease-related modifications in tau affect the interaction between Fyn and Tau. J. Biol. Chem. 280, 35119–35125. 10.1074/jbc.M505895200 16115884

[B5] Black M. M. (2016). Axonal transport: the orderly motion of axonal structures. Methods Cell Biol. 131, 1–19. 10.1016/bs.mcb.2015.06.001 26794507

[B6] Black M. M. Slaughter T. Moshiach S. Obrocka M. Fischer I. (1996). Tau is enriched on dynamic microtubules in the distal region of growing axons. J. Neurosci. 16, 3601–3619. 10.1523/JNEUROSCI.16-11-03601.1996 8642405 PMC6578833

[B7] Bliss T. V. Collingridge G. L. Morris R. G. Reymann K. G. (2018). Long-term potentiation in the hippocampus: discovery, mechanisms and function. Neuroforum 24, A103–A120. 10.1515/nf-2017-a059

[B9] Brady S. T. Morfini G. A. (2017). Regulation of motor proteins, axonal transport deficits and adult-onset neurodegenerative diseases. Neurobiol. Dis. 105, 273–282. 10.1016/j.nbd.2017.04.010 28411118 PMC5522763

[B8] Brady R. M. Zinkowski R. P. Binder L. I. (1995). Presence of tau in isolated nuclei from human brain. Neurobiol. Aging 16, 479–486. 10.1016/0197-4580(95)00023-8 7566354

[B10] Brandt R. Léger J. Lee G. (1995). Interaction of tau with the neural plasma membrane mediated by tau’s amino-terminal projection domain. J. Cell Biol. 131, 1327–1340. 10.1083/jcb.131.5.1327 8522593 PMC2120645

[B11] Brandt R. Trushina N. I. Bakota L. (2020). Much more than a cytoskeletal protein: physiological and pathological functions of the non-microtubule binding region of tau. Front. Neurol. 11:590059. 10.3389/fneur.2020.590059 33193056 PMC7604284

[B12] Buday L. Tompa P. (2010). Functional classification of scaffold proteins and related molecules. FEBS J. 277, 4348–4355. 10.1111/j.1742-4658.2010.07864.x 20883491

[B13] Carmel G. Mager E. M. Binder L. I. Kuret J. (1996). The structural basis of monoclonal antibody Alz50’s selectivity for Alzheimer’s disease pathology. J. Biol. Chem. 271, 32789–32795. 10.1074/jbc.271.51.32789 8955115

[B14] Carnegie G. K. Scott J. D. (2003). A-kinase anchoring proteins and neuronal signaling mechanisms. Genes Dev. 17, 1557–1568. 10.1101/gad.1095803 12842908

[B15] Chen S. Guttridge D. C. You Z. Zhang Z. Fribley A. Mayo M. W.. (2001). Wnt-1 signaling inhibits apoptosis by activating β-catenin/T cell factor-mediated transcription. J. Cell Biol. 152, 87–96. 10.1083/jcb.152.1.87 11149923 PMC2193656

[B16] Cleveland D. W. Hwo S. Y. Kirschner M. W. (1977a). Physical and chemical properties of purified tau factor and the role of tau in microtubule assembly. J. Mol. Biol. 116, 227–247. 10.1016/0022-2836(77)90214-5 146092

[B17] Cleveland D. W. Hwo S. Y. Kirschner M. W. (1977b). Purification of tau, a microtubule-associated protein that induces assembly of microtubules from purified tubulin. J. Mol. Biol. 116, 207–225. 10.1016/0022-2836(77)90213-3 599557

[B19] Combs B. Hamel C. Kanaan N. M. (2016). Pathological conformations involving the amino terminus of tau occur early in Alzheimer’s disease and are differentially detected by monoclonal antibodies. Neurobiol. Dis. 94, 18–31. 10.1016/j.nbd.2016.05.016 27260838 PMC4983528

[B18] Combs B. Kanaan N. M. (2017). Exposure of the amino terminus of tau is a pathological event in multiple tauopathies. Am. J. Pathol. 187, 1222–1229. 10.1016/j.ajpath.2017.01.019 28413156 PMC5818634

[B20] Cox K. Combs B. Abdelmesih B. Morfini G. Brady S. T. Kanaan N. M. (2016). Analysis of isoform-specific tau aggregates suggests a common toxic mechanism involving similar pathological conformations and axonal transport inhibition. Neurobiol. Aging 47, 113–126. 10.1016/j.neurobiolaging.2016.07.015 27574109 PMC5075521

[B21] Dawson H. N. Ferreira A. Eyster M. V. Ghoshal N. Binder L. I. Vitek M. P. (2001). Inhibition of neuronal maturation in primary hippocampal neurons from tau deficient mice. J. Cell Sci. 114, 1179–1187. 11228161 10.1242/jcs.114.6.1179

[B22] DiTella M. Feiguin F. Morfini G. Caceres A. (1994). Microfilament-associated growth cone component depends upon tau for its intracellular localization. Cell Motil. Cytoskeleton 29, 117–130. 10.1002/cm.970290204 7820862

[B23] Easton J. B. Royer A. R. Middlemas D. S. (2006). The protein tyrosine phosphatase, Shp2, is required for the complete activation of the RAS/MAPK pathway by brain-derived neurotrophic factor. J. Neurochem. 97, 834–845. 10.1111/j.1471-4159.2006.03789.x 16573649

[B24] El Khoury N. B. Gratuze M. Papon M. A. Bretteville A. Planel E. (2014). Insulin dysfunction and tau pathology. Front. Cell. Neurosci. 8:22. 10.3389/fncel.2014.00022 24574966 PMC3920186

[B25] Fá M. Puzzo D. Piacentini R. Staniszewski A. Zhang H. Baltrons M. A.. (2016). Extracellular tau oligomers produce an immediate impairment of LTP and memory. Sci. Rep. 6:19393. 10.1038/srep19393 26786552 PMC4726138

[B26] Fitzpatrick A. W. P. Falcon B. He S. Murzin A. G. Murshudov G. Garringer H. J.. (2017). Cryo-EM structures of tau filaments from Alzheimer’s disease. Nature 547, 185–190. 10.1038/nature23002 28678775 PMC5552202

[B27] Frandemiche M. L. De Seranno S. Rush T. Borel E. Elie A. Arnal I.. (2014). Activity-dependent tau protein translocation to excitatory synapse is disrupted by exposure to amyloid-β oligomers. J. Neurosci. 34, 6084–6097. 10.1523/JNEUROSCI.4261-13.2014 24760868 PMC6608293

[B28] Gibbs K. L. Greensmith L. Schiavo G. (2015). Regulation of axonal transport by protein kinases. Trends Biochem. Sci. 40, 597–610. 10.1016/j.tibs.2015.08.003 26410600

[B29] Goedert M. Spillantini M. G. (2019). Ordered assembly of tau protein and neurodegeneration. Adv. Exp. Med. Biol. 1184, 3–21. 10.1007/978-981-32-9358-8_1 32096024

[B30] Götz J. Xia D. Leinenga G. Chew Y. L. Nicholas H. (2013). What renders TAU toxic. Front. Neurol. 4:72. 10.3389/fneur.2013.00072 23772223 PMC3677143

[B31] Grant S. G. O’Dell T. J. Karl K. A. Stein P. L. Soriano P. Kandel E. R. (1992). Impaired long-term potentiation, spatial learning and hippocampal development in fyn mutant mice. Science 258, 1903–1910. 10.1126/science.1361685 1361685

[B32] Grundke-Iqbal I. Iqbal K. Quinlan M. Tung Y. C. Zaidi M. S. Wisniewski H. M. (1986). Microtubule-associated protein tau. A component of Alzheimer paired helical filaments. J. Biol. Chem. 261, 6084–6089. 10.1016/S0021-9258(17)38495-8 3084478

[B33] Gunasekaran K. Tsai C.-J. Kumar S. Zanuy D. Nussinov R. (2003). Extended disordered proteins: targeting function with less scaffold. Trends Biochem. Sci. 28, 81–85. 10.1016/S0968-0004(03)00003-3 12575995

[B34] Hagiwara M. Alberts A. Brindle P. Meinkoth J. Feramisco J. Deng T.. (1992). Transcriptional attenuation following cAMP induction requires PP-1-mediated dephosphorylation of CREB. Cell 70, 105–113. 10.1016/0092-8674(92)90537-m 1352481

[B35] Hanger D. P. Goniotaki D. Noble W. (2019). Synaptic localisation of tau. Adv. Exp. Med. Biol. 1184, 105–112. 10.1007/978-981-32-9358-8_9 32096032

[B36] Harada A. Oguchi K. Okabe S. Kuno J. Terada S. Ohshima T.. (1994). Altered microtubule organization in small-calibre axons of mice lacking tau protein. Nature 369, 488–491. 10.1038/369488a0 8202139

[B37] Hill E. Wall M. J. Moffat K. G. Karikari T. K. (2020). Understanding the pathophysiological actions of tau oligomers: a critical review of current electrophysiological approaches. Front. Mol. Neurosci. 13:155. 10.3389/fnmol.2020.00155 32973448 PMC7468384

[B38] Hoover B. R. Reed M. N. Su J. Penrod R. D. Kotilinek L. A. Grant M. K.. (2010). Tau mislocalization to dendritic spines mediates synaptic dysfunction independently of neurodegeneration. Neuron 68, 1067–1081. 10.1016/j.neuron.2010.11.030 21172610 PMC3026458

[B39] Horowitz P. M. Patterson K. R. Guillozet-Bongaarts A. L. Reynolds M. R. Carroll C. A. Weintraub S. T.. (2004). Early N-terminal changes and caspase-6 cleavage of tau in Alzheimer’s disease. J. Neurosci. 24, 7895–7902. 10.1523/JNEUROSCI.1988-04.2004 15356202 PMC6729917

[B40] Ihara Y. (1986). Alzheimer’s paired helical filaments. Rinsho Shinkeigaku 26, 1287–1289. 3103967

[B41] Ittner L. M. Ke Y. D. Delerue F. Bi M. Gladbach A. van Eersel J.. (2010). Dendritic function of tau mediates amyloid-β toxicity in Alzheimer’s disease mouse models. Cell 142, 387–397. 10.1016/j.cell.2010.06.036 20655099

[B42] Janning D. Igaev M. Sundermann F. Bruhmann J. Beutel O. Heinisch J. J.. (2014). Single-molecule tracking of tau reveals fast kiss-and-hop interaction with microtubules in living neurons. Mol. Biol. Cell 25, 3541–3551. 10.1091/mbc.E14-06-1099 25165145 PMC4230615

[B43] Jeganathan S. von Bergen M. Brutlach H. Steinhoff H. J. Mandelkow E. (2006). Global hairpin folding of tau in solution. Biochemistry 45, 2283–2293. 10.1021/bi0521543 16475817

[B44] Jicha G. A. Berenfeld B. Davies P. (1999). Sequence requirements for formation of conformational variants of tau similar to those found in Alzheimer’s disease. J. Neurosci. Res. 55, 713–723. 10.1002/(SICI)1097-4547(19990315)55:6<713::AID-JNR6>3.0.CO;2-G 10220112

[B45] Jicha G. A. Bowser R. Kazam I. G. Davies P. (1997). Alz-50 and MC-1, a new monoclonal antibody raised to paired helical filaments, recognize conformational epitopes on recombinant tau. J. Neurosci. Res. 48, 128–132. 10.1002/(sici)1097-4547(19970415)48:2<128::aid-jnr5>3.0.co;2-e 9130141

[B46] Kanaan N. M. Cox K. Alvarez V. E. Stein T. D. Poncil S. McKee A. C. (2016). Characterization of early pathological tau conformations and phosphorylation in chronic traumatic encephalopathy. J. Neuropathol. Exp. Neurol. 75, 19–34. 10.1093/jnen/nlv001 26671985 PMC4891281

[B47] Kanaan N. M. Morfini G. Pigino G. LaPointe N. E. Andreadis A. Song Y.. (2012). Phosphorylation in the amino terminus of tau prevents inhibition of anterograde axonal transport. Neurobiol. Aging 33, 826.e15–830.e15. 10.1016/j.neurobiolaging.2011.06.006 21794954 PMC3272324

[B48] Kanaan N. M. Morfini G. A. LaPointe N. E. Pigino G. F. Patterson K. R. Song Y.. (2011). Pathogenic forms of tau inhibit kinesin-dependent axonal transport through a mechanism involving activation of axonal phosphotransferases. J. Neurosci. 31, 9858–9868. 10.1523/JNEUROSCI.0560-11.2011 21734277 PMC3391724

[B49] Kanaan N. M. Pigino G. F. Brady S. T. Lazarov O. Binder L. I. Morfini G. A. (2013). Axonal degeneration in Alzheimer’s disease: when signaling abnormalities meet the axonal transport system. Exp. Neurol. 246, 44–53. 10.1016/j.expneurol.2012.06.003 22721767 PMC3465504

[B50] Kang S. Eskandari-Sedighi G. Hromadkova L. Safar J. Westaway D. (2020). Cellular biology of tau diversity and pathogenic conformers. Front. Neurol. 11:590199. 10.3389/fneur.2020.590199 33304310 PMC7693435

[B51] Keshet Y. Seger R. (2010). The MAP kinase signaling cascades: a system of hundreds of components regulates a diverse array of physiological functions. Methods Mol. Biol. 661, 3–38. 10.1007/978-1-60761-795-2_1 20811974

[B52] Kim Y. Liu G. Leugers C. J. Mueller J. D. Francis M. B. Hefti M. M.. (2019). Tau interacts with SHP2 in neuronal systems and in Alzheimer’s disease brains. J. Cell Sci. 132:jcs229054. 10.1242/jcs.229054 31201283 PMC6679582

[B53] Kimura T. Whitcomb D. J. Jo J. Regan P. Piers T. Heo S.. (2014). Microtubule-associated protein tau is essential for long-term depression in the hippocampus. Philos. Trans. R. Soc. Lond. B Biol. Sci. 369:20130144. 10.1098/rstb.2013.0144 24298146 PMC3843876

[B54] Klein C. Kramer E.-M. Cardine A.-M. Schraven B. Brandt R. Trotter J. (2002). Process outgrowth of oligodendrocytes is promoted by interaction of fyn kinase with the cytoskeletal protein tau. J. Neurosci. 22, 698–707. 10.1523/JNEUROSCI.22-03-00698.2002 11826099 PMC6758498

[B55] Kneynsberg A. Combs B. Christensen K. Morfini G. Kanaan N. M. (2017). Axonal degeneration in tauopathies: disease relevance and underlying mechanisms. Front. Neurosci. 11:572. 10.3389/fnins.2017.00572 29089864 PMC5651019

[B56] Kosik K. S. Orecchio L. D. Binder L. Trojanowski J. Q. Lee V. M. Lee G. (1988). Epitopes that span the tau molecule are shared with paired helical filaments. Neuron 1, 817–825. 10.1016/0896-6273(88)90129-8 2483104

[B57] LaPointe N. E. Morfini G. Pigino G. Gaisina I. N. Kozikowski A. P. Binder L. I.. (2009). The amino terminus of tau inhibits kinesin-dependent axonal transport: implications for filament toxicity. J. Neurosci. Res. 87, 440–451. 10.1002/jnr.21850 18798283 PMC2739042

[B58] Lau D. H. Hogseth M. Phillips E. C. O’Neill M. J. Pooler A. M. Noble W.. (2016). Critical residues involved in tau binding to fyn: implications for tau phosphorylation in Alzheimer’s disease. Acta Neuropathol. Commun. 4:49. 10.1186/s40478-016-0317-4 27193083 PMC4870772

[B59] Lebouvier T. Scales T. M. Hanger D. P. Geahlen R. L. Lardeux B. Reynolds C. H.. (2008). The microtubule-associated protein tau is phosphorylated by Syk. Biochim. Biophys. Acta 1783, 188–192. 10.1016/j.bbamcr.2007.11.005 18070606 PMC2258316

[B60] Lee G. Leugers C. J. (2012). Tau and tauopathies. Prog. Mol. Biol. Transl. Sci. 107, 263–293. 10.1016/B978-0-12-385883-2.00004-7 22482453 PMC3614411

[B61] Lee G. Newman S. T. Gard D. L. Band H. Panchamoorthy G. (1998). Tau interacts with src-family non-receptor tyrosine kinases. J. Cell Sci. 111, 3167–3177. 9763511 10.1242/jcs.111.21.3167

[B62] Lee G. Thangavel R. Sharma V. M. Litersky J. M. Bhaskar K. Fang S. M.. (2004). Phosphorylation of tau by fyn: implications for Alzheimer’s disease. J. Neurosci. 24, 2304–2312. 10.1523/JNEUROSCI.4162-03.2004 14999081 PMC6730442

[B63] Lee S.-H. Zabolotny J. M. Huang H. Lee H. Kim Y.-B. (2016). Insulin in the nervous system and the mind: functions in metabolism, memory, and mood. Mol. Metab. 5, 589–601. 10.1016/j.molmet.2016.06.011 27656397 PMC5021669

[B64] Lester L. B. Scott J. D. (1997). Anchoring and scaffold proteins for kinases and phosphatases. Recent Prog. Horm. Res. 52, 409–429; discussion 429–430. 9238861

[B66] Leugers C. J. Koh J. Y. Hong W. Lee G. (2013). Tau in MAPK activation. Front. Neurol. 4:161. 10.3389/fneur.2013.00161 24146661 PMC3797993

[B65] Leugers C. J. Lee G. (2010). Tau potentiates nerve growth factor-induced mitogen-activated protein kinase signaling and neurite initiation without a requirement for microtubule binding. J. Biol. Chem. 285, 19125–19134. 10.1074/jbc.M110.105387 20375017 PMC2885191

[B67] Li H.-L. Wang H.-H. Liu S.-J. Deng Y.-Q. Zhang Y.-J. Tian Q.. (2007). Phosphorylation of tau antagonizes apoptosis by stabilizing β-catenin, a mechanism involved in Alzheimer’s neurodegeneration. Proc. Natl. Acad. Sci. U S A 104, 3591–3596. 10.1073/pnas.0609303104 17360687 PMC1805527

[B68] Liao H. Li Y. Brautigan D. L. Gundersen G. G. (1998). Protein phosphatase 1 is targeted to microtubules by the microtubule-associated protein tau. J. Biol. Chem. 273, 21901–21908. 10.1074/jbc.273.34.21901 9705329

[B69] Liu C. W. Lee G. Jay D. G. (1999). Tau is required for neurite outgrowth and growth cone motility of chick sensory neurons. Cell Motil. Cytoskeleton 43, 232–242. 10.1002/(SICI)1097-0169(1999)43:3<232::AID-CM6>3.0.CO;2-7 10401579

[B70] Loomis P. A. Howard T. H. Castleberry R. P. Binder L. I. (1990). Identification of nuclear tau isoforms in human neuroblastoma cells. Proc. Natl. Acad. Sci. U S A 87, 8422–8426. 10.1073/pnas.87.21.8422 1700432 PMC54968

[B71] LoPresti P. (2002). Regulation and differential expression of tau mRNA isoforms as oligodendrocytes mature *in vivo*: implications for myelination. Glia 37, 250–257. 10.1002/glia.10035 11857683

[B72] LoPresti P. (2015). Inducible expression of a truncated form of tau in oligodendrocytes elicits gait abnormalities and a decrease in myelin: implications for selective CNS degenerative diseases. Neurochem. Res. 40, 2188–2199. 10.1007/s11064-015-1707-x 26394614

[B73] LoPresti P. (2018). Tau in oligodendrocytes takes neurons in sickness and in health. Int. J. Mol. Sci. 19:2408. 10.3390/ijms19082408 30111714 PMC6121290

[B74] Luo W. Peterson A. Garcia B. A. Coombs G. Kofahl B. Heinrich R.. (2007). Protein phosphatase 1 regulates assembly and function of the β-catenin degradation complex. EMBO J. 26, 1511–1521. 10.1038/sj.emboj.7601607 17318175 PMC1829374

[B75] Lüscher C. Malenka R. C. (2012). NMDA receptor-dependent long-term potentiation and long-term depression (LTP/LTD). Cold Spring Harb. Perspect. Biol. 4:a005710. 10.1101/cshperspect.a005710 22510460 PMC3367554

[B76] Maina M. B. Al-Hilaly Y. K. Serpell L. C. (2016). Nuclear tau and its potential role in Alzheimer’s disease. Biomolecules 6:9. 10.3390/biom6010009 26751496 PMC4808803

[B77] Mandelkow E. M. Mandelkow E. (2012). Biochemistry and cell biology of tau protein in neurofibrillary degeneration. Cold Spring Harb. Perspect. Med. 2:a006247. 10.1101/cshperspect.a006247 22762014 PMC3385935

[B78] Mandell J. W. Banker G. A. (1995). The microtubule cytoskeleton and the development of neuronal polarity. Neurobiol. Aging 16, 229–237; discussion 238. 10.1016/0197-4580(94)00164-v 7566333

[B79] Mansuroglu Z. Benhelli-Mokrani H. Marcato V. Sultan A. Violet M. Chauderlier A.. (2016). Loss of Tau protein affects the structure, transcription and repair of neuronal pericentromeric heterochromatin. Sci. Rep. 6:33047. 10.1038/srep33047 27605042 PMC5015075

[B80] Marciniak E. Leboucher A. Caron E. Ahmed T. Tailleux A. Dumont J.. (2017). Tau deletion promotes brain insulin resistance. J. Exp. Med. 214, 2257–2269. 10.1084/jem.20161731 28652303 PMC5551570

[B81] McInnes J. Wierda K. Snellinx A. Bounti L. Wang Y.-C. Stancu I.-C.. (2018). Synaptogyrin-3 mediates presynaptic dysfunction induced by tau. Neuron 97, 823.e8–835.e8. 10.1016/j.neuron.2018.01.022 29398363

[B82] Mermoud J. E. Cohen P. Lamond A. I. (1992). Ser/Thr-specific protein phosphatases are required for both catalytic steps of pre-mRNA splicing. Nucleic Acids Res. 20, 5263–5269. 10.1093/nar/20.20.5263 1331983 PMC334330

[B83] Min S.-W. Chen X. Tracy T. E. Li Y. Zhou Y. Wang C.. (2015). Critical role of acetylation in tau-mediated neurodegeneration and cognitive deficits. NatMed 21, 1154–1162. 10.1038/nm.3951 26390242 PMC4598295

[B84] Miyamoto T. Stein L. Thomas R. Djukic B. Taneja P. Knox J.. (2017). Phosphorylation of tau at Y18, but not tau-fyn binding, is required for tau to modulate NMDA receptor-dependent excitotoxicity in primary neuronal culture. Mol. Neurodegener. 12:41. 10.1186/s13024-017-0176-x 28526038 PMC5438564

[B85] Moreno H. Morfini G. Buitrago L. Ujlaki G. Choi S. Yu E.. (2016). Tau pathology-mediated presynaptic dysfunction. Neuroscience 325, 30–38. 10.1016/j.neuroscience.2016.03.044 27012611 PMC4887082

[B90] Morfini G. A. Burns M. Binder L. I. Kanaan N. M. LaPointe N. Bosco D. A.. (2009). Axonal transport defects in neurodegenerative diseases. J. Neurosci. 29, 12776–12786. 10.1523/JNEUROSCI.3463-09.2009 19828789 PMC2801051

[B86] Morfini G. Schmidt N. Weissmann C. Pigino G. Kins S. (2016). Conventional kinesin: biochemical heterogeneity and functional implications in health and disease. Brain Res. Bull. 126, 347–353. 10.1016/j.brainresbull.2016.06.009 27339812

[B87] Morfini G. Szebenyi G. Brown H. Pant H. C. Pigino G. DeBoer S.. (2004). A novel CDK5-dependent pathway for regulating GSK3 activity and kinesin-driven motility in neurons. EMBO J. 23, 2235–2245. 10.1038/sj.emboj.7600237 15152189 PMC419914

[B88] Morfini G. Szebenyi G. Elluru R. Ratner N. Brady S. T. (2002). Glycogen synthase kinase 3 phosphorylates kinesin light chains and negatively regulates kinesin-based motility. EMBO J. 21, 281–293. 10.1093/emboj/21.3.281 11823421 PMC125832

[B89] Morfini G. Szebenyi G. Richards B. Brady S. T. (2001). Regulation of kinesin: implications for neuronal development. Dev. Neurosci. 23, 364–376. 10.1159/000048720 11756752

[B92] Morris M. Maeda S. Vossel K. Mucke L. (2011). The many faces of tau. Neuron 70, 410–426. 10.1016/j.neuron.2011.04.009 21555069 PMC3319390

[B93] Morris S. L. Tsai M.-Y. Aloe S. Bechberger K. Konig S. Morfini G.. (2020). Defined tau phosphospecies differentially inhibit fast axonal transport through activation of two independent signaling pathways. Front. Mol. Neurosci. 13:610037. 10.3389/fnmol.2020.610037 33568975 PMC7868336

[B94] Müller C. Bauer N. M. Schäfer I. White R. (2013). Making myelin basic protein-from mRNA transport to localized translation. Front. Cell. Neurosci. 7:169. 10.3389/fncel.2013.00169 24098271 PMC3784684

[B95] Nakazawa T. Komai S. Tezuka T. Hisatsune C. Umemori H. Semba K.. (2001). Characterization of Fyn-mediated tyrosine phosphorylation sites on GluR epsilon 2 (NR2B) subunit of the N-methyl-D-aspartate receptor. J. Biol. Chem. 276, 693–699. 10.1074/jbc.M008085200 11024032

[B96] Ondrejcak T. Klyubin I. Corbett G. T. Fraser G. Hong W. Mably A. J.. (2018). Cellular prion protein mediates the disruption of hippocampal synaptic plasticity by soluble tau *in vivo*. J. Neurosci. 38, 10595–10606. 10.1523/JNEUROSCI.1700-18.2018 30355631 PMC6290298

[B97] Ott A. Stolk R. P. van Harskamp F. Pols H. A. Hofman A. Breteler M. M. (1999). Diabetes mellitus and the risk of dementia: the Rotterdam study. Neurology 53, 1937–1942. 10.1212/wnl.53.9.1937 10599761

[B98] Padmanabhan P. Martínez-Mármol R. Xia D. Götz J. Meunier F. A. (2019). Frontotemporal dementia mutant Tau promotes aberrant Fyn nanoclustering in hippocampal dendritic spines. eLife 8:e45040. 10.7554/eLife.45040 31237563 PMC6592683

[B99] Papasozomenos S. C. Binder L. I. (1987). Phosphorylation determines two distinct species of Tau in the central nervous system. Cell Motil. Cytoskeleton 8, 210–226. 10.1002/cm.970080303 2446784

[B100] Patterson K. R. Ward S. M. Combs B. Voss K. Kanaan N. M. Morfini G.. (2011). Heat shock protein 70 prevents both tau aggregation and the inhibitory effects of preexisting tau aggregates on fast axonal transport. Biochemistry 50, 10300–10310. 10.1021/bi2009147 22039833 PMC3387688

[B101] Pérez M. J. Jara C. Quintanilla R. A. (2018). Contribution of tau pathology to mitochondrial impairment in neurodegeneration. Front. Neurosci. 12:441. 10.3389/fnins.2018.00441 30026680 PMC6041396

[B102] Puzzo D. Piacentini R. Fá M. Gulisano W. Li Puma D. D. Staniszewski A.. (2017). LTP and memory impairment caused by extracellular Aβ and Tau oligomers is APP-dependent. eLife 6:e26991. 10.7554/eLife.26991 28696204 PMC5529106

[B103] Rapoport M. Dawson H. N. Binder L. I. Vitek M. P. Ferreira A. (2002). Tau is essential to β -amyloid-induced neurotoxicity. Proc. Natl. Acad. Sci. U S A 99, 6364–6369. 10.1073/pnas.092136199 11959919 PMC122954

[B104] Ratner N. Bloom G. S. Brady S. T. (1998). A role for cyclin-dependent kinase(s) in the modulation of fast anterograde axonal transport: effects defined by olomoucine and the APC tumor suppressor protein. J. Neurosci. 18, 7717–7726. 10.1523/JNEUROSCI.18-19-07717.1998 9742142 PMC6793030

[B105] Rebelo S. Santos M. Martins F. da Silva E. F. E. da Cruz e Silva O. A. (2015). Protein phosphatase 1 is a key player in nuclear events. Cell. Signal. 27, 2589–2598. 10.1016/j.cellsig.2015.08.007 26275498

[B106] Regan P. Piers T. Yi J.-H. Kim D.-H. Huh S. Park S. J.. (2015). Tau phosphorylation at serine 396 residue is required for hippocampal LTD. J. Neurosci. 35, 4804–4812. 10.1523/JNEUROSCI.2842-14.2015 25810511 PMC4389589

[B107] Reynolds C. H. Garwood C. J. Wray S. Price C. Kellie S. Perera T.. (2008). Phosphorylation regulates tau interactions with Src homology 3 domains of phosphatidylinositol 3-kinase, phospholipase Cγ1, Grb2, and Src family kinases. J. Biol. Chem. 283, 18177–18186. 10.1074/jbc.M709715200 18467332

[B108] Roberson E. D. Scearce-Levie K. Palop J. J. Yan F. Cheng I. H. Wu T.. (2007). Reducing endogenous tau ameliorates amyloid β-induced deficits in an Alzheimer’s disease mouse model. Science 316, 750–754. 10.1126/science.1141736 17478722

[B109] Roche K. W. Standley S. McCallum J. Dune Ly C. Ehlers M. D. Wenthold R. J. (2001). Molecular determinants of NMDA receptor internalization. Nat. Neurosci. 4, 794–802. 10.1038/90498 11477425

[B110] Rong Y. Lu X. Bernard A. Khrestchatisky M. Baudry M. (2001). Tyrosine phosphorylation of ionotropic glutamate receptors by Fyn or Src differentially modulates their susceptibility to calpain and enhances their binding to spectrin and PSD-95. J. Neurochem. 79, 382–390. 10.1046/j.1471-4159.2001.00565.x 11677266

[B111] Rovelet-Lecrux A. Lecourtois M. Thomas-Anterion C. Le Ber I. Brice A. Frebourg T.. (2009). Partial deletion of the MAPT gene: a novel mechanism of FTDP-17. Hum. Mutat. 30, E591–E602. 10.1002/humu.20979 19263483

[B112] Sabbagh J. J. Dickey C. A. (2016). The metamorphic nature of the tau protein: dynamic flexibility comes at a cost. Front. Neurosci. 10:3. 10.3389/fnins.2016.00003 26834532 PMC4720746

[B113] Seiberlich V. Bauer N. G. Schwarz L. Ffrench-Constant C. Goldbaum O. Richter-Landsberg C. (2015). Downregulation of the microtubule associated protein tau impairs process outgrowth and myelin basic protein mRNA transport in oligodendrocytes. Glia 63, 1621–1635. 10.1002/glia.22832 25847153

[B114] Sengupta S. Horowitz P. M. Karsten S. L. Jackson G. R. Geschwind D. H. Fu Y.. (2006). Degradation of tau protein by puromycin-sensitive aminopeptidase *in vitro*. Biochemistry 45, 15111–15119. 10.1021/bi061830d 17154549

[B115] Sharma V. M. Litersky J. M. Bhaskar K. Lee G. (2007). Tau impacts on growth-factor-stimulated actin remodeling. J. Cell Sci. 120, 748–757. 10.1242/jcs.03378 17284520

[B116] Shaw A. S. Filbert E. L. (2009). Scaffold proteins and immune-cell signalling. Nat. Rev. Immunol. 9, 47–56. 10.1038/nri2473 19104498

[B117] Sinsky J. Majerova P. Kovac A. Kotlyar M. Jurisica I. Hanes J. (2020). Physiological tau interactome in brain and its link to tauopathies. J. Proteome Res. 19, 2429–2442. 10.1021/acs.jproteome.0c00137 32357304

[B118] Smith F. D. Langeberg L. K. Scott J. D. (2006). The where’s and when’s of kinase anchoring. Trends Biochem. Sci. 31, 316–323. 10.1016/j.tibs.2006.04.009 16690317

[B119] Song Y. Kang M. Morfini G. Brady S. T. (2016). Fast axonal transport in isolated axoplasm from the squid giant axon. Methods Cell Biol. 131, 331–348. 10.1016/bs.mcb.2015.07.004 26794522

[B120] Sontag E. Nunbhakdi-Craig V. Lee G. Brandt R. Kamibayashi C. Kuret J.. (1999). Molecular interactions among protein phosphatase 2A, tau, and microtubules. Implications for the regulation of tau phosphorylation and the development of tauopathies. J. Biol. Chem. 274, 25490–25498. 10.1074/jbc.274.36.25490 10464280

[B121] Sontag J.-M. Nunbhakdi-Craig V. White C. L. III. Halpain S. Sontag E. (2012). The protein phosphatase PP2A/Bα binds to the microtubule-associated proteins tau and MAP2 at a motif also recognized by the kinase Fyn: implications for tauopathies. J. Biol. Chem. 287, 14984–14993. 10.1074/jbc.M111.338681 22403409 PMC3340226

[B122] Sotiropoulos I. Galas M. C. Silva J. M. Skoulakis E. Wegmann S. Maina M. B.. (2017). Atypical, non-standard functions of the microtubule associated tau protein. Acta Neuropathol. Commun. 5:91. 10.1186/s40478-017-0489-6 29187252 PMC5707803

[B123] Spillantini M. G. Goedert M. (1998). Tau protein pathology in neurodegenerative diseases. Trends Neurosci. 21, 428–433. 10.1016/s0166-2236(98)01337-x 9786340

[B124] Stern J. L. Lessard D. V. Ali R. Berger C. L. (2017). Single-molecule imaging of tau dynamics on the microtubule surface. Methods Cell Biol. 141, 135–154. 10.1016/bs.mcb.2017.06.016 28882299 PMC9848482

[B125] Sun J. Nan G. (2017). The extracellular signal-regulated kinase 1/2 pathway in neurological diseases: a potential therapeutic target (Review). Int. J. Mol. Med. 39, 1338–1346. 10.3892/ijmm.2017.2962 28440493 PMC5428947

[B126] Sun W. Qureshi H. Y. Cafferty P. W. Sobue K. Agarwal-Mawal A. Neufield K. D.. (2002). Glycogen synthase kinase-3β is complexed with tau protein in brain microtubules. J. Biol. Chem. 277, 11933–11940. 10.1074/jbc.M107182200 11812770

[B127] Suzuki T. Okumura-Noji K. (1995). NMDA receptor subunits epsilon 1 (NR2A) and epsilon 2 (NR2B) are substrates for Fyn in the postsynaptic density fraction isolated from the rat brain. Biochem. Biophys. Res. Commun. 216, 582–588. 10.1006/bbrc.1995.2662 7488151

[B128] Tai C. Chang C.-W. Yu G. Q. Lopez I. Yu X. Wang X.. (2020). Tau reduction prevents key features of autism in mouse models. Neuron 106, 421.e11–437.e11. 10.1016/j.neuron.2020.01.038 32126198 PMC7210056

[B129] Talbot K. Wang H.-Y. Kazi H. Han L. Y. Bakshi K. P. Stucky A.. (2012). Demonstrated brain insulin resistance in Alzheimer’s disease patients is associated with IGF-1 resistance, IRS-1 dysregulation, and cognitive decline. J. Clin. Invest. 122, 1316–1338. 10.1172/JCI59903 22476197 PMC3314463

[B130] Thurston V. C. Zinkowski R. P. Binder L. I. (1996). Tau as a nucleolar protein in human nonneural cells *in vitro* and *in vivo*. Chromosoma 105, 20–30. 10.1007/BF02510035 8662255

[B131] Tiernan C. T. Combs B. Cox K. Morfini G. Brady S. T. Counts S. E.. (2016). Pseudophosphorylation of tau at S422 enhances SDS-stable dimer formation and impairs both anterograde and retrograde fast axonal transport. Exp. Neurol. 283, 318–329. 10.1016/j.expneurol.2016.06.030 27373205 PMC4992631

[B132] Tompa P. Fersht A. (2009). Structure and Function of Intrinsically Disordered Proteins. New York, NY: CRC Press.

[B133] Tracy T. E. Gan L. (2018). Tau-mediated synaptic and neuronal dysfunction in neurodegenerative disease. Curr. Opin. Neurobiol. 51, 134–138. 10.1016/j.conb.2018.04.027 29753269 PMC6130905

[B134] Tracy T. E. Sohn P. D. Minami S. S. Wang C. Min S. W. Li Y.. (2016). Acetylated tau obstructs kibra-mediated signaling in synaptic plasticity and promotes tauopathy-related memory loss. Neuron 90, 245–260. 10.1016/j.neuron.2016.03.005 27041503 PMC4859346

[B135] Trushina N. I. Bakota L. Mulkidjanian A. Y. Brandt R. (2019). The evolution of tau phosphorylation and interactions. Front. Aging Neurosci. 11:256. 10.3389/fnagi.2019.00256 31619983 PMC6759874

[B136] Umemori H. Wanaka A. Kato H. Takeuchi M. Tohyama M. Yamamoto T. (1992). Specific expressions of Fyn and Lyn, lymphocyte antigen receptor-associated tyrosine kinases, in the central nervous system. Mol. Brain Res. 16, 303–310. 10.1016/0169-328x(92)90239-8 1337939

[B137] Uversky V. N. (2015). Intrinsically disordered proteins and their (disordered) proteomes in neurodegenerative disorders. Front. Aging Neurosci. 7:18. 10.3389/fnagi.2015.00018 25784874 PMC4345837

[B138] Voskas D. Ling L. S. Woodgett J. R. (2010). Does GSK-3 provide a shortcut for PI3K activation of Wnt signalling? F1000 Biol. Rep. 2:82. 10.3410/B2-82 21283602 PMC3026644

[B139] Warmus B. A. Sekar D. R. McCutchen E. Schellenberg G. D. Roberts R. C. McMahon L. L.. (2014). Tau-mediated NMDA receptor impairment underlies dysfunction of a selectively vulnerable network in a mouse model of frontotemporal dementia. J. Neurosci. 34, 16482–16495. 10.1523/JNEUROSCI.3418-14.2014 25471585 PMC4252555

[B140] Weingarten M. D. Lockwood A. H. Hwo S. Y. Kirschner M. W. (1975). A protein factor essential for microtubule assembly. Proc. Natl. Acad. Sci. U S A 72, 1858–1862. 10.1073/pnas.72.5.1858 1057175 PMC432646

[B141] Weissmann C. Reyher H.-J. Gauthier A. Steinhoff H. J. Junge W. Brandt R. (2009). Microtubule binding and trapping at the tip of neurites regulate tau motion in living neurons. Traffic 10, 1655–1668. 10.1111/j.1600-0854.2009.00977.x 19744140

[B142] White R. Gonsior C. Kramer-Albers E. M. Stohr N. Huttelmaier S. Trotter J. (2008). Activation of oligodendroglial Fyn kinase enhances translation of mRNAs transported in hnRNP A2-dependent RNA granules. J. Cell Biol. 181, 579–586. 10.1083/jcb.200706164 18490510 PMC2386098

[B143] Witman G. B. Cleveland D. W. Weingarten M. D. Kirschner M. W. (1976). Tubulin requires tau for growth onto microtubule initiating sites. Proc. Natl. Acad. Sci. U S A 73, 4070–4074. 10.1073/pnas.73.11.4070 1069293 PMC431332

[B144] Wolozin B. L. Pruchnicki A. Dickson D. W. Davies P. (1986). A neuronal antigen in the brains of Alzheimer patients. Science 232, 648–650. 10.1126/science.3083509 3083509

[B145] Wood J. G. Mirra S. S. Pollock N. J. Binder L. I. (1986). Neurofibrillary tangles of Alzheimer disease share antigenic determinants with the axonal microtubule-associated protein tau (tau). Proc. Natl. Acad. Sci. U S A 83, 4040–4043. 10.1073/pnas.83.11.4040 2424015 PMC323661

[B146] Xia D. Li C. Götz J. (2015). Pseudophosphorylation of Tau at distinct epitopes or the presence of the P301L mutation targets the microtubule-associated protein Tau to dendritic spines. Biochim. Biophys. Acta 1852, 913–924. 10.1016/j.bbadis.2014.12.017 25558816

[B147] Yoshiyama Y. Higuchi M. Zhang B. Huang S. M. Iwata N. Saido T. C.. (2007). Synapse loss and microglial activation precede tangles in a P301S tauopathy mouse model. Neuron 53, 337–351. 10.1016/j.neuron.2007.01.010 17270732

[B148] Zhao X. Kotilinek L. A. Smith B. Hlynialuk C. Zahs K. Ramsden M.. (2016). Caspase-2 cleavage of tau reversibly impairs memory. Nat. Med. 22, 1268–1276. 10.1038/nm.4199 27723722

[B149] Zhong H. Sia G.-M. Sato T. R. Gray N. W. Mao T. Khuchua Z.. (2009). Subcellular dynamics of type II PKA in neurons. Neuron 62, 363–374. 10.1016/j.neuron.2009.03.013 19447092 PMC2702487

[B150] Zhou L. McInnes J. Wierda K. Holt M. Herrmann A. G. Jackson R. J.. (2017). Tau association with synaptic vesicles causes presynaptic dysfunction. Nat. Commun. 8:15295. 10.1038/ncomms15295 28492240 PMC5437271

